# WHO, WHEN, HOW: a scoping review on flexible at-home respite for informal caregivers of older adults

**DOI:** 10.1186/s12913-024-11058-0

**Published:** 2024-06-26

**Authors:** Maude Viens, Alexandra Éthier, Véronique Provencher, Annie Carrier

**Affiliations:** 1 Université de Sherbrooke, Sherbrooke, Québec Canada; 2 Research Center on Aging, Sherbrooke, Québec Canada

**Keywords:** Respite, Home care/homecare, Informal caregiving, Older adults, Scoping review

## Abstract

**Background:**

As the world population is aging, considerable efforts need to be put towards developing and maintaining evidenced-based care for older adults. Respite services are part of the selection of homecare offered to informal caregivers. Although current best practices around respite are rooted in person centeredness, there is no integrated synthesis of its flexible components. Such a synthesis could offer a better understanding of key characteristics of flexible respite and, as such, support its implementation and use.

**Methods:**

To map the literature around the characteristics of flexible at-home respite for informal caregivers of older adults, a scoping study was conducted. Qualitative data from the review was analyzed using content analysis. The characterization of flexible at-home respite was built on three dimensions: *WHO*, *WHEN* and *HOW*. To triangulate the scoping results, an online questionnaire was distributed to homecare providers and informal caregivers of older adults.

**Results:**

A total of 42 documents were included in the review. The questionnaire was completed by 105 participants. The results summarize the characteristics of flexible at-home respite found in the literature. Flexibility in respite can be understood through three dimensions: (1) *WHO* is tendering it, (2) *WHEN* it is tendered and (3) *HOW* it is tendered. Firstly, human resources (*WHO*) must be compatible with the homecare sector as well as being trained and qualified to offer respite to informal caregivers of older adults. Secondly, flexible respite includes considerations of time, duration, frequency, and predictability (*WHEN*). Lastly, flexible at-home respite exhibits approachability, appropriateness, affordability, availability, and acceptability (*HOW*). Overall, flexible at-home respite adjusts to the needs of the informal caregiver and care recipient in terms of *WHO*, *WHEN*, and *HOW*.

**Conclusion:**

This review is a step towards a more precise definition of flexible at-home respite. Flexibility of homecare, in particular respite, must be considered when designing, implementing and evaluating services.

## Background

It is an undeniable fact that the world population is aging [1]. The World Health Organization [1] estimates that from 2015 to 2050, the percentage of people over 60 years of age will nearly double (from 12 to 22%). Governments must therefore put in place policies, laws and funding infrastructures to provide evidence-based social services and healthcare that are in line with best practices to allow people to age in place [2]. Aging in place refers to “the ability to live in one’s own home and community safely, independently, and comfortably, regardless of age, income, or ability level” [3]. Relevant literature indicates that people do not want to age or end their lives in institutionalized care; most wish to receive care in their home and remain in their community with their informal caregivers [4].

There is then a need to adequately support informal caregivers (caregiver) in the crucial role that they have in allowing older adults to age in their own home. A caregiver is “a person who provides some type of unpaid, ongoing assistance with activities of daily living or instrumental activities of daily living” [5]. In their duties, caregivers of older adults are responsible for a considerable amount of homecare [6]: Transportation, management of appointments and bills, domestic chores, etc. Private and public organizations offer a plethora of services to support caregivers of older adults (e.g., support groups, housekeeping, etc.), including respite. Respite is a service for caregivers consisting in “the temporary provision of care for a person, at home or in an institution, by people other than the primary caregiver” [7]. Maayan and collaborators [7] characterize all respite services according to three dimensions: (1) *WHERE*: The place; in a private home, a daycare centre or a residential setting, (2) *WHEN*: The duration and planning; ranging from a couple of hours to a number of weeks, planned or unplanned, and finally, (3) *WHO*: The person providing the service; this may be trained or untrained individuals, paid staff or volunteers. Respite is widely recognized as necessary to support caregivers of older adults [8, 9]. Indeed, a large number of studies identify the need and use for respite [9–12]. For example, Dal Santo and colleagues (2007) found that caregivers of older adults (*n* = 1643) used respite to manage stressful caregiving situations, but also to have a “time away”, without having to worry about their caregiving role [13]. At-home respite seems to be favoured over other forms of respite, even with the perceived drawbacks, such as the privacy breach of having a care worker in one’s home [14, 15].

Studies suggest that caregivers of older adults seek flexibility as a main component of respite [16–18]. Flexibility, in line with person-centered care, allows respite that addresses their needs, rather than being services that are prescribed according to other criteria [16, 17]. Thus, flexibility, both in accessing and in the respite itself, is essential [19–23]. Although there seems to be a consensus around the broader definition of respite, there is no literature reviewing the characteristics of flexible at-home respite. Some studies and reports from organizations and governments document the flexible characteristics of their models, but there are few literature reviews that address them, specifically [18, 22, 24]. Both reviews by Shaw et al. [18] and Neville et al. [19] concede that an operational definition of respite (*WHEN*, *WHERE*, *WHO*) is not clear. Neville et al. [19] conclude that “respite has the potential to be delivered in flexible and positive ways”, without addressing these ways. The absence of a unified definition for flexible at-home respite contributes to the challenges of implementing and evaluating services, as well as measuring their effect. Although respite services are deemed necessary, they are seldom used [19, 25–27]; as little as 6% of all caregivers receiving any kind support services in Canada actually use them. In scientific literature, the under-usage of respite services is a shared reality around the world [28]. One of the main reasons for this under-usage is the overall lack of flexibility in both obtaining and using respite [29, 30]. Synthesizing the characteristics of flexible at-home respite services is the first steppingstone to a common operational definition. This could contribute to increasing respite use through the implementation or enrichment of programs in ways that answer the dyad’s (caregiver and older adult) needs.

Consequently, to support the implementation and evaluation of homecare programs, the objective of this study was to synthesize the knowledge on the characteristics of flexible at-home respite services offered to caregivers of older adults.

## Method

A scoping review [32–34] was conducted, as part of a larger multi-method participatory research known as the AMORA project [31] to characterize flexible at-home respite. Scoping reviews allow to map the extent of literature on a specific topic [32, 34]. The six steps proposed by Levac et al. [32] were followed: [1] Identifying the research question; [2] searching and [3] selecting pertinent documents; [4] extracting (*or charting*) relevant data; [5] collating, summarizing and reporting findings; [6] consultation with stakeholders. The sixth step is optional.

### Identifying the research question

The research question was: “What are the characteristics of flexible at-home respite services offered to caregivers of older adults?” As the research was conducted, this question was divided into three sub-questions:

1. *WHO* is tendering flexible respite?
2. *WHEN* is flexible respite tendered?
3. *HOW* is flexible respite tendered?

### Identifying relevant documents

The search strategy consisted of two methods. First, the key words (1) respite (2) informal caregivers (3) older adults in the title or abstract allowed to identify relevant documents (Table 1). Initially included, the term “flexib*” was removed from the search, given the low number generated (60 versus 1,179 documents without). The first author and a librarian specialized in health sciences research documentation conducted the literature research in July of 2021 and updated it in December of 2022 in 6 databases (*Ageline*, *Cochrane*, *CINAHL*, *Medline*, *PsychInfo*, and *Abstracts in Social Gerontology*). The expanded research strategy then consisted of the identification of relevant documents from the selected bibliography and one article that was found by searching for unavailable references (alternative article).

**Table 1:** Database searches and keywords used

	Respite for caregivers of OLDER ADULTS
Databases (*n* = 6)	Ageline, Cochrane, CINAHL, Medline, PsychInfo, ASG
Keywords (*n* = 11)	1. **Respite** TI (respite) OR AB (respite) OR SU (respite) 2. **Informal caregiver** TI (caregiver* OR carer* OR “natural helper*” OR “care giver*” OR spouse* OR husband*) OR AB (caregiver* OR carer* OR “natural helper*” OR “care giver*” OR spouse* OR husband*) OR SU (caregiver* OR carer* OR “natural helper*” OR “care giver*” OR spouse* OR husband*) 3. **Senior** TI (aged OR elder* OR senior* OR “older people” OR “older adult*” OR “older person*” OR geriatr* OR “fragile adult*” OR ageing OR aging OR dementia* OR alzheimer*) OR AB (aged OR elder* OR senior* OR “older people” OR “older adult*” OR “older person*” OR geriatr* OR “fragile adult*” OR ageing OR aging OR dementia* OR alzheimer*) OR SU (aged OR elder* OR senior* OR “older people” OR “older adult*” OR “older person*” OR geriatr* OR “fragile adult*” OR ageing OR aging OR dementia* OR alzheimer*)

### Study selection

**Fig. 1:**
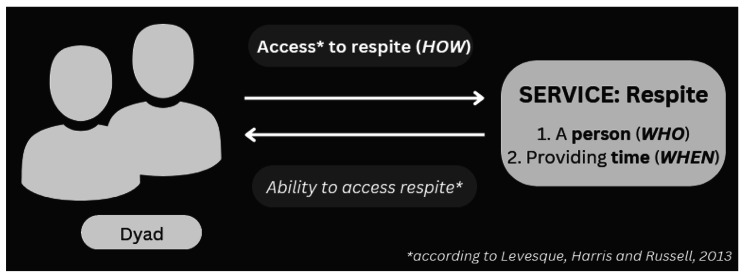
Conceptual mapping of results: *HOW*, *WHEN*, *WHO*

To review the most recent literature on flexible at-home respite service characteristics, the research team focused on writings within a 20-year span, as have other reviews (e.g., [35, 36]); documents thus had to be published between 2001 and 2022. The research team selected documents written in French or English, only. Included documents had to come from either (1) scientific literature (i.e., articles in an academic journal presenting an empirical study or reviews) or (2) reports and briefs from government, homecare organizations or research centres. All study designs were included. The research team convened that at-home respite is an (1) individual (i.e., not in a group) service (although, theoretically, two persons living in the same household could receive it) from (2) a professional or a volunteer that occurs (3) in the home and that (4) it requires no transport for the dyad. To select documents related to flexible at-home respite, the research team identified those in which the respite displayed an ability to adapt to the dyad’s needs on at least one characteristic of the service, as presented by Maayan and collaborators (*WHERE[Not relevant to this review, as it focuses on at-home respite]*, *WHO*, *WHEN*). The team concluded that these three dimensions lacked the precision to globally characterize the service. Indeed, they did not describe access to or activities occurring during respite, or, as the team called it, the *HOW* (Fig. 1). Excluded documents were those covering several services at once, preventing the differentiation of elements that were specific to at-home respite services. As this is a scoping review, the research team did not include a critical appraisal of individual sources of evidence [32, 34].

Following the step-by-step Preferred Reporting Items for Systematic reviews and Meta-Analyses extension for Scoping Reviews (PRISMAScR) guidelines [37], the research team met to define the selection strategy. First, they screened the documents by their titles and abstracts, before determining their eligibility, based on their full text. Considering the limited human and financial resources, at each step of the PRISMAScR, a second team member assessed 10% of the documents independently to co-validate the selection; the goal was to reach 80% of agreement between both team members regarding document inclusion or exclusion. If an agreement was not reached, they would meet to obtain a consensus. The research team used *Zotero* reference management software to store documents as well as a cloud-based website to collaborate on the selection.

### Charting the data

The first author charted (or extracted) both quantitative and qualitative data. To quantitatively characterize documents, contextual data (country of origin, year of publication, type of documents, etc.) was extracted in a *Microsoft Excel* table. For the qualitative data, the research team created an extraction table in *Microsoft Word* that included the three dimensions of respite (*WHO*, *WHEN* and *HOW)* and one “*other*” dimension, as to not force any excerpts under the three dimensions. To co-validate the data charting, the second and third authors replicated 10% of the process. Expressly, the first author extracted elements related to a flexible characteristic of the at-home respite (*WHO*, *WHEN*, *HOWorother*). Considering limited resources, the third and second authors both co-validated the extraction of 10% of the documents. Authors met to reach a consensus where a disagreement arose.

### Collating, summarizing, and reporting the results

**Fig. 2:**
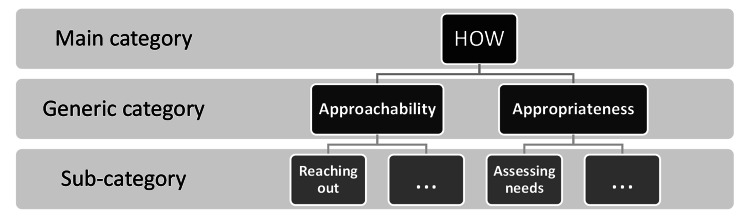
Content analysis: Types of categories according to Elo and Kyngäs (2007) (*with examples from results*)

The research team used content analysis to “attain a condensed and broad description of the phenomenon” [38]. To do so, data was prepared (familiarization with the data and extraction of pertinent excerpts) and organized (classification of excerpts) to build a characterization of flexible at-home respite. In this scoping review, a deductive content analysis began with three main categories (*WHO*, *WHEN*, *HOW*), with the addition of the temporary “*other*” category. Content analysis aimed to divide these categories into several generic categories, which subdivided into sub-categories (Fig. 2), inductively. This allowed to define the three main categories. While the *WHO* and the *WHEN* categories describe the service itself (time, duration, qualified staff, etc.), the *HOW* category is specific to the interface between the organization offering respite and the dyad (assessing the needs of the dyad, coordinating care, etc.). An interface is a situation where two “subjects” interact and affect each other [39]. In the context of homecare services, Levesque, Harris and Russell (2013) have defined that interface as access [40]. Therefore, to define the generic categories of the *HOW*, the team used the five dimensions of their access to care framework: Approachability, appropriateness, affordability, availability and acceptability [40]. Approachability relates to users recognizing the existence and accessibility of a service [40]. Appropriateness encompasses the alignment between services and users’ needs, considering timeliness and assessment of needs [40]. Affordability pertains to users’ economic capacity to allocate resources for accessing suitable services [40]. Availability signifies that services can be reached, both physically and in a timely manner [40]. Acceptability involves cultural and social factors influencing users’ willingness to accept services [40]. In other words, the *HOW* category focuses on the organizational or professional aspects of the service and how they can be adapted to the dyad.

To co-validate the classification, the research team met until they were all satisfied with the categorization. The first author then completed the classification. After classifying 20% of the documents, the second author would comment the classification. When the authors reached an agreement, the first author would move on to the classification of another 20%. First and second authors would meet when disagreements about classification and categories arose, to confer and adjust. Finally, all categories were discussed with the third author, until a consensus was reached. Once categorization was achieved, the team prepared a synthesis report. In this report, the team defined the main categories (*WHO*, *WHEN, HOW*, *other*) and their generic and sub-categories (Fig. 2) with pertinent excerpts from the reviewed literature. In summary, the results of the scoping review characterize flexible at-home respite under three attributes: *WHO*, *WHEN* and *HOW*.

### Consultation

Rather than conducting a focus group as suggested by Levac and collaborators [32], the team chose to triangulate the results with those from a survey as a consultation strategy. Specifically, the research team took advantage of a survey being conducted with relevant stakeholders in the larger study (AMORA project), as it allowed to respect the scoping review’s allocated resources. The survey aimed to define flexible at-home respite and the factors affecting its implementation and delivery. A committee including a researcher, a doctoral student and a representative of an organization funding homecare services in Québec (Canada), developed the survey following the three stages proposed by Corbière and Fraccaroli [41]. It originally included a total of 21 items: Thirteen [13] close-ended and 8 open-ended questions. Of these 8, 2 addressed the characteristics of an ideal at-home service and suggestions regarding respite and were used here for triangulation purposes. The questionnaire was published online, in French, on the *Microsoft Forms*® platform in the summer of 2020. Recruitment of participants (caregivers and people from the homecare sector) was done via email, by contacting regional organizations (Eastern Townships, Québec, Canada). In addition, the 18 senior consultation tables spread throughout the territory of the province of Québec were solicited; working in collaboration with governmental instances in charge of services to older adults and caregivers, these tables bring together representatives for associations, groups or organizations concerned with their living conditions.

The goal was to triangulate the scoping review’s results, i.e., to identify what was common between the literature and real-world experiences, and, as such, to bring contextual value to the results. Accordingly, the team analyzed data using mixed categorization [42]. The categories from the scoping review served as a starting point (closed categorization), leaving room to create new categories, as the analysis progressed (open categorization). Once all the data (scoping and survey) was categorized, the team identified the characteristics according to sources. To do so, the team tabulated the reoccurrence of each category in the survey, in the scoping review, or in both. They then integrated the results to provide one unified categorization of flexible at-home respite. The AMORA project was approved by the research ethics committee of the Integrated University Health and Social Services Centre (CIUSSS) of the Eastern Townships (project number: 2021–3703).

## Results

**Fig. 3:**
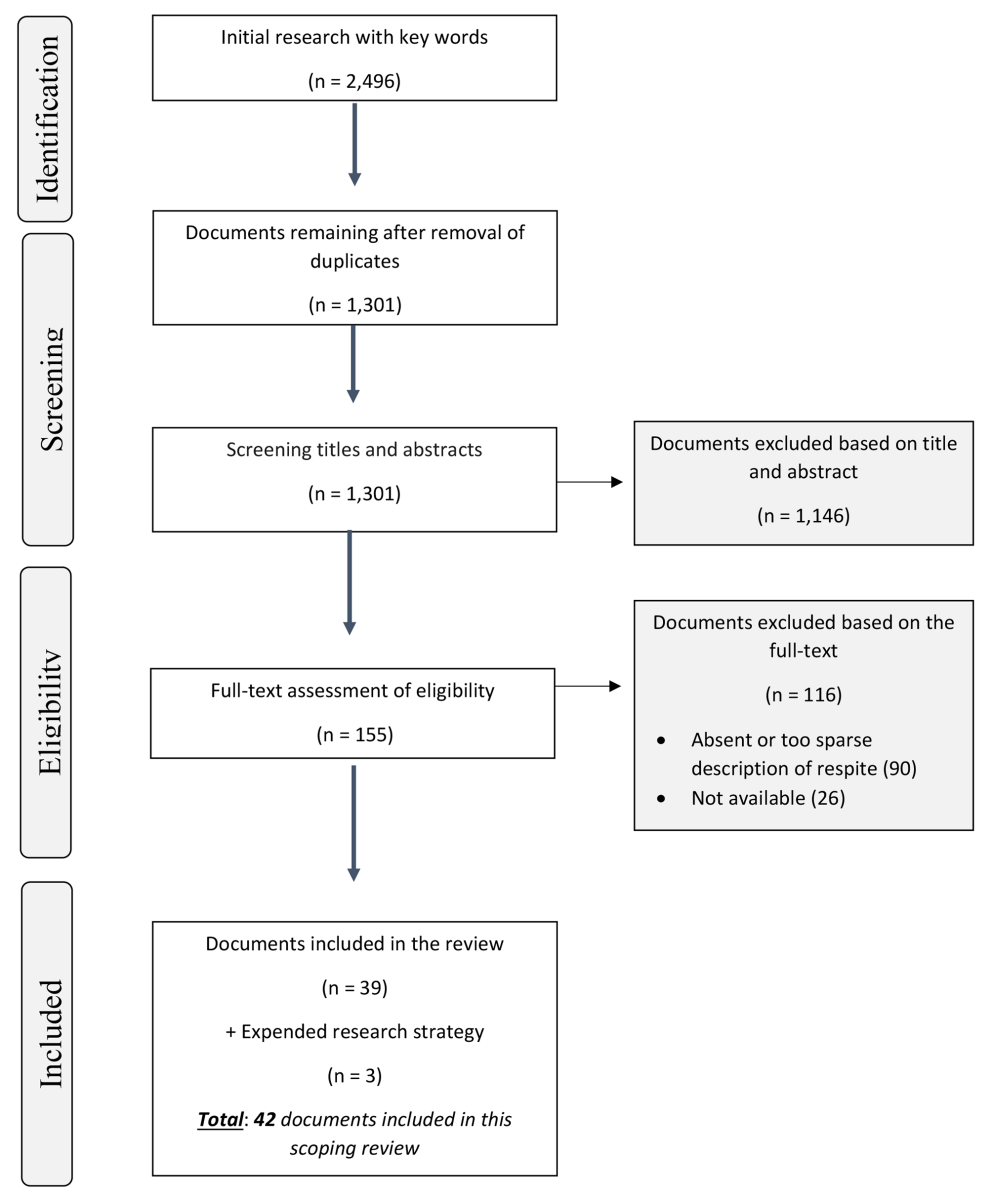
Preferred Reporting Items for Systematic reviews and Meta-Analyses extension for Scoping Reviews (PRISMAScR) flow chart of the scoping review process [37]

Of the 1,301 papers retrieved through the database searches, 1,146 were not eligible based on title and abstract, while 116 were excluded after reading their full texts, resulting in 39 included documents (Fig. 3). Documents were mainly excluded because they did not provide details about the respite service and its flexibility. The expanded search yielded three additional documents, resulting in a total of 42 documents, included in this scoping review. This section details (1) the characteristics of the selected documents and (2) the characterization of flexible at-home respite.

### Characteristics of selected documents

The majority (86%) of the documents in the review (Table 2) are from after 2005, with only 14% of the documents published before 2005, and are from 9 countries; United States (*n* = 18; 42%), United Kingdom (*n* = 11; 26%), Australia (*n* = 4; 10%), Canada (*n* = 2; 5%), Ireland (*n* = 2; 5%), France (*n* = 2; 5%), Belgium (*n* = 2; 5%), Germany (*n* = 1; 2%), New Zealand (*n* = 1). The types of documents were diverse: 68% (*n* = 28) were empirical studies, 31% (*n* = 13) theoretical papers and 1% (*n* = 2) government briefs. Most (*n* = 23; 56%) of the documents did not specify their research approach, while 10 and 9 took, respectively, a qualitative (23%) or quantitative approach (21%). Most documents address respite in the context of caregiving for someone living with Alzheimer’s disease or other neurocognitive disorders (*n* = 25; 60%), while some targeted older adults in general (*n* = 14; 34%), people in palliative care (*n* = 4; 9%) or other older adult populations (for example, veterans) (*n* = 3; 1%). Respite was usually tendered by community organizations specialized in homecare (*n* = 32; 78%). Although the majority of the documents (*n* = 31; 75%) did not address the type of region (rural, urban, or mixed) surrounding the caregivers, those who did (*n* = 11; 26%) mainly reported being in a mixed environment (*n* = 9; 21%).

### Characteristics of survey participants

Although all 100 participants completed the questionnaire, 71 participants answered at least 1 of the 2 open-ended questions: Each question had 66 and 41 answers. Of those 71 participants, most of them were women (*n* = 60; 85%). All participants were aged on average 55 years old (SD = 15). They were mostly from the Eastern Townships area (*n* = 56; 79%). Most participants were either caregivers (*n* = 24; 34%) or homecare workers (*n* = 28; 39%), while some were service administrators (*n* = 11; 15%), and some reported being both caregivers as well as working in the formal caregiving sector (*n* = 7; 10%). Only one person reported themselves as an older adult having a caregiver.

**Table 2:** Description of selected studies; authors, country, year, type of documents & characteristics of flexible respite in text

				Characteristics		
Authors	Country	Year	Type of document	*WHEN*	*WHO*	*HOW*
Administration for Community Living [43]	U.S.	n.a.	Government brief			X
Arksey et al. [44]	U.K.	2004	Empirical study (mixed)	X	X	X
Barrett et al. [45]	Aus.	2009	Empirical study (mixed)		X	X
Bayly et al. [46]	Canada	2020	Empirical study (quali.)	X		X
Bun & Baker [47]	U.K.	2006	Theoretical article	X		X
Caulfield, Seddon, Williams, & Hedd Jones [48]	U.K.	2021	Empirical study (quali.)			X
Derence [49]	U.S.	2005	Theoretical article			X
Evans & Lee [50]	Aus.	2013	Empirical study (quanti.)	X	X	X
Feinberg [51]	U.S.	2006	Government brief			X
Feinberg & Newman [24]	U.S.	2006	Empirical study (mixt.)			X
Fox [52]	U.K.	2011	Theoretical article	X		X
Gendron & Adam [53]	Canada	2005	Empirical study (quali.)	X	X	X
Hesse [54]	Germany	2005	Empirical study (quanti.)		X	X
Hopkinson et al. [55]	U.K.	2021	Empirical study (mixed)	X		X
Ingleton, Payne, Nolan & Carey [56]	U.K.	2003	Empirical study (quali.)	X		
Kelly & Williams [57]	U.S.	2007	Empirical study (quali.)			X
King & Parsons [58]	New Zea.	2005	Empirical study (mixed)			X
Kristjanson et al. [59]	Aus.	2004	Empirical study (mixed)		X	X
LaVela, Johnson, Miskevics, & Weaver [60]	U.S.	2012	Empirical study (quanti.)	X		X
Link [61]	U.S.	2016	Theoretical article			X
Lucet [62]	France	2015	Theoretical article	X	X	X
Marquant [63]	France	2010	Theoretical article		X	X
Mason et al. [64]	U.K.	2007	Empirical study (quanti.)	X		X
McKay, Taylor & Armstrong [65]	Ireland	2013	Empirical study (mixed)	X		X
Moriarty [66]	U.K.	2002	Theoretical article	X		X
Noelker & Browdie [67]	U.S.	2012	Theoretical article			X
Parahoo, Campbell & Scoltock [68]	Ireland	2002	Empirical study (quali.)	X	X	X
Perks et al. [69]	U.K.	2001	Theoretical article	X	X	X
Rosenthal Gelman et al. [70]	U.S.	2014	Empirical study (mixed)		X	X
Ryan, Noble, Thorpe & Nolan [14]	U.K.	2008	Theoretical article	X	X	X
Shanley [17]	Aus.	2006	Empirical study (quali.)	X	X	X
Shaw et al. [18]	U.K.	2009	Empirical study (mixed)	X	X	X
Smith [71]	U.S.	2007	Empirical study (quanti.)			X
Sorrell [72]	U.S.	2006	Theoretical article	X		
Staicovici [73]	U.S.	2003	Theoretical article	X		X
Starns, Karner & Montgomery [74]	U.S.	2002	Empirical study (quali.)			X
Swartzell, Fulton & Crowder [75]	U.S.	2022	Empirical study (quanti.)			X
Tompkins & Bell [76]	U.S.	2009	Empirical study (quanti.)			X
Vandepitte et al. [77]	Belgium	2019	Empirical study (quanti.)	X		X
Vandepitte et al. [21]	Belgium	2016	Empirical study (quanti.)	X	X	X
Washington & Tachman [78]	U.S.	2017	Empirical study (quali.)	X	X	X
Whitlatch & Feinberg [79]	U.S.	2006	Empirical study (quanti.)			X
*Total per main categories*				23	16	40

### Characterization of flexible at-home respite

The characterization of flexible at-home respite will be presented below in three main categories which are *WHO*, *WHEN*, and *HOW*. Of note, 10 (24%) of the included documents had three categories of flexible components, 16 (38%) had 2 categories and 1 category. Almost all documents discussed the *HOW* of flexible at-home respite (*n* = 40, 95%). Out of the 33 categories constructed with the scoping review, only 6 (18%) were not reported in the questionnaire: (1) planned respite (*WHEN*), (2) screening of dyads (*HOW*), (3) determining frequency of respite (*HOW*), (4) coordination of care (*HOW*), (5) voucher approach (*HOW*) and (6) acceptability to low-income households (*HOW*). Moreover, the questionnaire added three characteristics that were not present in the scoping review: (1) respite needs to be approachable, (2) the organization must be prompt** and adhocratic** and (3) able to deliver respite regardless of the season** (availability). Generic or sub-categories present only in the scoping review are identified with 1 asterisk (*), while those present only in the questionnaire have 2 (**).

### WHO

In the selected documents, the *WHO* dimension of flexible at-home respite services can be broken down into three qualifiers: (1) *Compatible*, (2) *qualified* and (3) *trained* (Table 3). This dimension includes all human resources contributing to homecare (administrative staff, governing bodies, paid and volunteer care workers). First, the workforce behind flexible respite is *compatible*, meaning it has personal characteristics and profiles relevant to homecare for caregivers of older adults [17, 53, 62, 63, 68]. Gendron and Adam explain this by describing how the role of the care worker in Baluchon Alzheimer™ goes beyond training: “The nature of their work with [Baluchon Alzheimer™] requires particular human and professional qualities that are quite as important as academic credentials” [53]. Personal characteristics such as flexibility [53, 62, 63, 68], empathy and patience [17, 53, 62] are deemed essential attributes. Secondly, the workforce is *qualified*: It has the necessary skills, abilities and knowledge from past professional [14, 45, 62, 70] and personal experience [62] to work, or volunteer, with caregivers of older adults. For a program like Baluchon Alzheimer™, “the backgrounds of the *baluchonneuses* vary […]; all have experience in gerontology” [53]. Other areas of qualification in the included documents are a nursing background [18, 45] or knowledge related to dementia [69]. Finally, flexible at-home respite requires a *trained* workforce engaged in the process of acquiring knowledge and learning the skills to provide respite services to caregivers of older adults. For example, homecare organizations can offer specific training on various topics, depending on their target clientele: Dementia [44], palliative care [59], or homecare in general [44].

**Table 3:** Summary – Characterization of flexible at-home respite: *WHO* [14, 17, 18, 21, 44, 45, 50, 53, 54, 59, 62, 63, 68–70, 78]

Characteristics	Definition
Compatible [17, 53, 62, 63, 68]	Personal characteristics and profile relevant to respite: For example, flexibility and empathy
Qualified [14, 18, 45, 50, 53, 62, 69, 70]	Skills, abilities, and knowledge relevant to respite from professional and personal experiences
Trained [14, 17, 21, 44, 53, 54, 59, 62, 63, 78]	Knowledge and skills relevant to respite acquired from their homecare organization

### WHEN

The *WHEN* dimension of flexible at-home respite contains 4 temporal features: (1) *Time*, (2) *duration*, (3) *frequency* and (4) *predictability* (Table 4). First, flexible respite is available on a wide range of possible *time* slots. For example, the service is “available 24 hours, but typically from 9 am to 10 pm” [64]. Secondly, flexible respite is accessible on a wide range of possible *durations*. The Community Dementia Support Service (CDSS) is an example of flexibility in duration by “[being] totally flexible, being available from 2 to 15 hours per week” [69]. Thirdly, the service is offered in different *frequencies*: It can be either recurrent or occasional, or a combination of both [18, 64, 66]. The last feature of the *WHEN* dimension is flexibility in *predictability*; the respite service can be planned* or not. A study on respite services in South Australia found that most providers (93%) planned the respite care with the dyad, but that emergency or crisis services were still offered by 35% of them [50].

**Table 4:** Summary – Characterization of flexible at-home respite: *WHEN* [14, 17, 18, 21, 44, 46, 50, 52–56, 59, 60, 62, 64–66, 68, 69, 72, 73, 77, 78]

Characteristics	Definition
Time [14, 18, 44, 46, 52, 56, 60, 64–66, 68, 69, 72]	Wide range of time slots
Duration [14, 21, 44, 53, 60, 62, 64, 65, 68, 69, 72, 73, 77]	Wide range of service duration
Frequency [17, 18, 55, 64, 66, 72]	Recurrent or occasional/punctual service
Predictability [17, 18, 44, 50, 54, 55, 59, 64–66, 78]	Planned or not, or both
• Planned* [17, 50, 54, 59]	Can be planned
• Not planned [18, 44, 50, 55, 64–66, 78]	Can be an “emergency” or “crisis” intervention

Legend: *: Generic or sub-category present only in the scoping review

### HOW

At-home respite is flexible when it demonstrates *approachability*: Caregivers can identify that some form of respite exists and can be reached (Table 5). For the respite service to be approachable, the organization needs to be reaching out to dyads; it proactively makes sure that caregivers of older adults have information on services, know of their existence and that they can be used. For example, the El Portal program put in place “advisory groups that included the local clergy, representatives from businesses, caregivers, and service providers who were used for outreach work” [66]. The organization also screens* dyads to assess their eligibility for respite, as well as for other services from the same program or organization. For example, the North Carolina (U.S.A.) Project C.A.R.E. has an initial assessment that considers the range of homecare services available, rather than just assessing for eligibility for a program [57]. In addition, flexible respite requires the organization to set attainable and inclusive requirements for eligibility, as to not discourage use [24, 57, 61, 66]. Finally, the organization communicates consistently with the dyad. As Shanley explains in their literature review, “there are clear and open ways for carers to express concerns about the service, and an open mechanism is available for dealing with these concerns constructively” [17]. In addition, the survey participants discussed two other characteristics. First, for respite to be approachable, the organization is prompt**, respecting a reasonable delay between the request and the beginning of the service (wait list). Second, it is adhocratic**, meaning the organization does not depend on complex systems of rules and procedures to operate i.e., bureaucracy.

**Table 5:** Summary – Characterization of flexible at-home respite: Approachability [17, 21, 24, 48, 53, 57, 61, 65, 66, 69, 70, 73, 77]

Characteristics	Definition
	The organization …
Reaching out [48, 65, 66, 69, 70, 73]	…proactively ensures that dyads have information on services.
Screening* [17, 57]	…assesses whether a dyad is eligible for the respite services, but also for other services.
Setting requirements [24, 57, 61, 66, 75]	…has set attainable and inclusive requirements for eligibility.
Communicating [17, 21, 53, 77]	…communicates consistently with the dyad.
Prompt**	…respects a reasonable delay between the request and the beginning of the service.
Adhocratic**	…does not depend on a complex system of rules and procedures.

Legend: *: Generic or sub-category present only in the scoping review. **: Generic or sub-category present only in the questionnaire

The second access dimension of flexible at-home respite is *appropriateness* (Table 6): The fit between respite services and the dyad’s needs, its timeliness, the amount of care spent in assessing their needs and determining the correct respite service. For the respite service to be appropriate, the organization assesses needs by collecting details about the dyad’s needs; this can include, but is not limited to, clinical, psychological, or social evaluation. The organization then proposes respite services from a wide range of options or packages: A multi-respite package, as presented by Arksey et al., can simply be the combination of at least two different respite services [44]. For the service to be appropriate, the organization also paces the respite. Apprehension towards service appropriateness can be mitigated by a gradual introduction to homecare, for example when the respite is presented as a trial [68]. The organization determines the service with the dyad and defines its different characteristics (WHEN*, *WHO*) so interventions correspond to their needs. The organization then determines the appropriate activities to do with the dyad during the respite. For example, the caregiver of older adults can be encouraged to use respite time for leisure (sleep, physical activity, etc.) [45], while the care worker supports the beneficiary in engaging in an activity such as a walk or a board game [14]. Furthermore, the organization coordinates* the services for the dyad and acts as a “respite broker” to arrange all aspects of care; this is especially relevant for programs that include a “care budget” that can be used at the caregivers’ discretion [58]. Finally, for the respite to be appropriate, the organization assures that it is in continuity with other health services, by connecting the dyads to pertinent resources. As described by Shaw, respite should be “embedded in a context that includes assessment, carer education, case management and counselling” [18].

**Table 6:** Summary – Characterization of flexible at-home respite: Appropriateness [14, 17, 18, 21, 43–55, 57–60, 62–71, 73, 77, 78]

*Characteristics*	Definition
	The organization…
Assessing needs [17, 44, 49, 51, 53–55, 59, 62, 67, 69, 70, 78]	…collects details about their clients’ needs (care recipient, caregiver and/or dyad).
Proposing respite [17, 18, 44, 46, 50, 51, 57, 58, 64, 66, 67, 71, 73, 75]	…offers respite services from a wide range of respite options or multi-respite packages.
Pacing respite [17, 21, 53, 55, 57, 60, 65, 68, 78]	…introduces respite services to the dyad progressively.
Determining respite [14, 17, 18, 21, 44, 45, 47, 50, 52–55, 57, 59, 62, 63, 65, 68, 69, 77, 78]	… determines the characteristics of the services with the dyad: *WHEN* & *WHO*
• WHEN [17, 18, 44, 45, 50, 54, 65, 68, 78]	… determines the temporal aspects of the respite with the dyad:
o Time [17, 44, 45, 50]	The time the services take place according to their needs.
o Duration [44, 45, 50]	The duration of the services according to their needs.
o Frequency* [18, 44, 50, 68]	The frequency of services according to their needs.
o Predictability [17, 50, 54, 65, 78]	The level of predictability of services according to their needs.
• WHO [14, 17, 18, 21, 44, 47, 50, 52, 53, 55, 57, 59, 62, 63, 65, 68, 69, 77, 78]	… determines the human resource dimension with the dyad. The workforce is…
o Stability [14, 18, 44, 47, 52, 53, 62, 68, 69, 78]	… constant.
o Responsiveness [14, 17, 18, 21, 44, 50, 53, 55, 57, 59, 62, 63, 65, 68, 69, 77]	… able to adapt to users’ expectations, values, and rights.
Determining activities [14, 17, 18, 44, 45, 47, 50, 52–55, 65, 68, 69, 78]	… determines with the dyad the activities to do during the respite.
Coordinating care* [18, 43, 44, 49, 51, 54, 55, 57, 58, 64, 70, 78]	… manages the respite services for the dyad.
Continuing care [17, 18, 44, 48, 49, 53, 55, 57, 58, 64, 65, 67, 69, 77]	… assures that the respite services are in continuity with other services.

Legend: *: Generic or sub-category present only in the scoping review

The third access dimension of flexible at-home respite is *affordability*, referring to the economic capacity of the dyad to spend resources to use appropriate respite services (Table 7). The included documents only explored the direct cost of respite: The amount of money a dyad must pay to receive services. For the respite to be affordable, its direct cost is either (1) adapted, where the cost is modulated according to the dyad’s financial resources, for example on a sliding scale, based on income or (2) nonexistent [44].

**Table 7:** Summary - Characterization of flexible at-home respite: Affordability [44, 52–54, 64, 78]

Characteristics	Definition
Direct cost [44, 52–54, 64, 78]	The amount of money a dyad must pay to receive respite services:
• Adapted [44, 52–54, 64]	Modulated according to the dyad’s financial resources
• Inexistent [44, 78]	No direct cost

Next, flexible at-home respite must demonstrate *availability* (Table 8): Services can be reached both physically and in a timely manner. Firstly, the organization offers respite in the dyads’ geographic area. Shanley described an at-home mobile respite program designed to reach rural and remote areas, where two care workers visit different locations for set periods of time [17]. Moreover, one sub-characteristic identified exclusively by the survey participants was seasonality. Indeed, the dyad has access to respite, regardless of the season**. Thus, the geography category is broken down between the access to service (1) in rural or remote areas and (2) notwithstanding the season. Flexibility in availability also requires that the dyads have access to unlimited respite time; the organization does not assign a finite bank of hours. Finally, the organization proposes diverse payment methods to the dyads. The consumer-directed approach is a way that homecare organizations offer flexibility. A care budget is allocated to the caregiver to purchase hours from homecare agencies or to hire their own respite workers. This includes payments to family members or friends to provide respite care [79]. An example of a type of consumer-directed approach is the use of vouchers*: Credit notes or coupons to purchase service hours from homecare agencies [44].

**Table 8:** Summary - Characterization of flexible at-home respite: Availability [17, 18, 24, 44, 46, 49, 51, 57, 64, 71, 76, 79]

Characteristics	Definition
	The organization offers…
Geography [17, 75]	… services in the dyad’s geographic area.
• Rurality [17, 75]	… services in rural and remote areas.
• Seasonality**	… services regardless of the season.
Time [18, 44]	… unlimited respite time.
Payment methods [18, 24, 44, 46, 49, 51, 57, 64, 71, 76, 79]	… different methods of payment.
• Consumer-directed [18, 24, 44, 46, 49, 51, 57, 64, 71, 72, 76, 79]	A care budget to purchase hours from homecare agencies or to hire respite workers
o Voucher* [18, 24, 44, 46, 51, 71, 76, 79]	A credit note to purchase service hours from homecare agencies

Legend: *: Generic or sub-category present only in the scoping review. **: Generic or sub-category present only in the questionnaire

Finally, access to flexible at-home respite also relates to *acceptability* (Table 9): The cultural and social factors determining the possibility for the dyad to accept respite and the perception of the appropriateness of seeking services. For the respite to be acceptable, the organization targets and caters to the cultural diversity represented in their local population. The organization is also able to identify and to accommodate underserved groups. In the included documents, underserved groups lacked access to respite for two reasons: (1) Geographic isolation or (2) the requirements to be eligible to “traditional homecare” does not apply to them, for example, for younger people with dementia and people with HIV/AIDS [17]. The organization can target and cater to low-income households*. Rosenthal Gelman and his collaborators detail a program where, after realizing that low-income caregivers have greater unmet needs, special funds were set aside for respite care vouchers to be distributed [70].

**Table 9:** Summary - Characterization of flexible at-home respite: Acceptability [17, 18, 44, 49, 57, 60, 66, 68, 70, 74]

Characteristics	Definition
	The organization…
Cultural diversity [17, 66, 70, 74]	… can target and cater to the different cultures represented in their local population.
Underserved groups [17, 18, 49, 57, 60, 68]	… can target and cater to underserved groups in their local population.
Low income households* [44, 57, 70]	… can target and cater to low-income households.

Legend: *: Generic or sub-category present only in the scoping review. **: Generic or sub-category present only in the questionnaire

## Discussion

This scoping review conducted with Levac and colleagues’ method [32] synthesized the knowledge on the characteristics of flexible at-home respite services offered to caregivers of older adults, from 42 documents. The results provide a synthesis of the characteristics of flexible at-home respite discussed in the literature. The three dimensions of flexibility in respite relate to (1) *WHO* is tendering it, (2) *WHEN* it is tendered and (3) *HOW* it is tendered. First, human resources (*WHO*) must be compatible with the homecare sector as well as being trained and qualified to offer respite to caregivers of older adults. The second feature of flexible respite is temporality (*WHEN*): The time, duration, frequency, and predictability of the service. The last dimension, access (*HOW*), refers to the interface between the respite and the users. Flexible at-home respite exhibits approachability, appropriateness, affordability, availability, and acceptability. In the light of what we learned, flexible at-home respite could be characterized as a service that has the ability to adjust to the needs of the dyad on all three dimensions (**W***HO*, *WHEN*, *HOW*). However, this seems to be more of an ideal than a reflection of reality.

The survey provided complementary results to the review; the concordance between the two is strong (27/33 = 82%). Six [6] characteristics were missing from the survey results, including planned respite and the voucher approach (*HOW*). Moreover, the survey added three elements to the review results: The organization’s adhocracy (*HOW*) and promptness (*HOW*) as well as its ability to offer services, regardless of the season (*HOW*). These mismatches might reflect the Québec (and possibly Canadian) landscape of homecare. For example, in the Québec homecare system, respite is mostly planned, it is therefore not surprising that people only mention that unplanned respite is lacking. The “voucher system” was not mentioned in the survey, probably in part because it does not exist in the province of Québec. Additionally, navigating the healthcare system to have free or affordable homecare can be treacherous [80]. In short, older adults have to go through (1) evaluation(s) by a social worker from a hospital or another public healthcare organization and (2) various administrative tasks (*adhocratic*) [2], before possibly being put on a waiting list (*prompt*) [81]. In addition, Canada can experience harsh winters (*seasonality*) that can make transport, which is an integral part of homecare, particularly laborious. Although those categories could reflect the particularity of homecare in Canada, a promising follow up on this review would be to compare the characteristics of flexible respite from one territory to another. It would contribute to providing a more operational definition of flexible at-home respite.

The remainder of this discussion will focus on two main points before touching on the limitations and strengths of this review. First, flexibility in at-home respite seems exceptional. Second, respite care workers are as skilled as they are underappreciated.

This review, in coherence with the literature, highlights the fact that respite services generally lack flexibility: This is the conclusion of several studies on respite [7, 64, 82]. A pattern seems to emerge in the countries represented in the review: Community organizations specialized in homecare (public and/or privately funded) offer respite on predetermined time slots, usually prescribed between traditional office hours (9 AM to 6 PM) [50]. This lack of flexibility could be explained in part by the rigidity of the structure of homecare services and the fact that its funding does not allow for customizable and punctual services [17, 62, 73]. Nevertheless, there were some examples of flexible respite models, such as Baluchon Alzheimer™ and consumer-directed approaches. Baluchon Alzheimer™ offers long-term at-home respite (4 to 14 days) by qualified and trained *baluchonneuses*. Prior to the relay of the caregivers, the baluchonneuse takes the time to learn about the dyad, including their environment and routine [53, 62]. Caregivers report feeling refreshed upon their return and appreciate the diaries (or logbooks) that the *baluchonneuse* meticulously fills out [53]. Another example would be consumer-directed approaches, where caregivers are attributed a budget to hire their own care worker. Allowing caregivers to choose their care worker (either from a self-employed carer or family and friends) can increase the quality of care and satisfaction, while providing relatively affordable care, especially in a situation of labour shortage [51, 79]. Even though these two models are a demonstration of how respite can be adapted to the caregiver-senior dyad, for the most part, flexibility is lacking on all three dimensions of respite (*WHO*, *WHEN*, *HOW*).

Secondly, the results from the scoping review highlight how homecare as a profession is often overlooked. Indeed, the reviewed documents state the necessary set of skills to offer respite; the level described is one of highly specialized care professionals with important liability. These skills must also transcend advanced knowledge and qualifications, to include interpersonal capabilities [17, 53, 62, 63, 68]. Furthermore, care workers must also be flexible to offer a wide range of service time and duration, in addition to being ready to provide “on-the-go” respite [53, 68]. Yet, the occupation of homecare worker is an underappreciated and underpaid position [83]. Community care, like respite, is generally not a priority for social and healthcare funding [24]. This can be explained in part by the neoliberal approach to care in which the target is to minimize spending and maximize (measurable) outcomes [84]. Homecare outcomes are often overlooked in favour of service delivery evaluation, in part because they are difficult to measure [44]. This approach can also lead to prioritizing third party contracting instead of including respite in the range of public services, as to save on expenses related to employment (insurance and other benefits) [85]. Another contributor is that funding is used for service administration and not to adequately provide services or remunerate care workers [86]. Finally, care workers are mostly women, known for doing the invisible work that is at the heart of respite care (emotional support, etc.) [87]. A telling example from the reviewed documents is that Baluchon Alzheimer™ refers to their care workers as baluchonneuses (feminine form) and not baluchoneurs (masculine form) [53]. Consequently, the homecare sector is faced with recruitment and retention challenges [44, 64, 88]. Authors of the documents included in the review addressed the fact that flexibility in service meant that service providers had to function with excess capacity; for example, by building an “employee bank” to cover all the hours of the day and emergency calls [44]. Ultimately, staff turnover and shortage caused in part by the work being underappreciated could create a vicious cycle, leading to inflexibility in respite. In short, overlooking and underestimating the crucial and specialized work of homecare workers can contribute to staff turnover, which in turn could result in a lack of flexibility of at-home respite.

### Limitations and strengths

The review’s methodological approach has some limitations and strengths. First, according to Levac, Colquhoun and O’Brien [32], research teams could conduct a sixth step in their scoping study, consisting in consulting experts through a focus group or workshop. This last phase aims at providing further insight into the review’s results and to begin the knowledge translation process. The team did not conduct a traditional consultation phase. Instead, they triangulated the review’s results through a questionnaire. This method was of interest, because of the natural concordance between the results and the considerable number of participants (*n* = 100). The survey still allowed to refine the characterization of respite, but further knowledge transfer to homecare actors and caregivers is necessary. Although innovative, there is a need to further investigate the validity of this approach as a consultation phase. Secondly, the theme of flexible at-home respite may have narrowed the search and identification of relevant documentation, and therefore caused the team to overlook some of the literature. Empirical studies and reviews on respite seldom include a detailed description of services [89–91]. This made it challenging to understand what services are like, operationally, for the dyad and to judge their flexibility. In addition, it complexified the extraction of relevant data, as descriptions were sparse and scattered throughout the documents. The team worked to mitigate these limitations in the documentation research and data charting phase. To begin, they sorted through all the literature on at-home respite for caregivers of older adults. In other words, the team not only searched for, but also included any explicit mention of flexibility. After selection, the extraction tables allowed enough versatility to include all the flexible characteristics of service, regardless of their placement in the text (introduction, methodology or discussion) or length. Another limitation is that, due to resource constraints, only 10% of the document selection and extraction was assessed by two reviewers, although a minimum of 80% of agreement was met and discussions were used to reach consensus where a disagreement arose. To conclude, strengths of this review include the extensiveness and diversity of the documents and its rigorous methodology, co-validated by a peer and an experienced researcher, with assistance from a specialized librarian.

## Conclusion

This review has both scientific and practical implications. From a scientific point of view, the results contribute to the body of knowledge on flexible respite service models for caregivers of seniors, an under-documented topic. To our knowledge, this is the first review that aims to characterize flexible at-home respite. Our results suggest the relevance of further documenting the factors influencing the implementation and delivery of flexible respite services, as well as the consequences of the lack of flexibility in respite services, which may lead to service underuse. Moreover, researchers could focus on documenting respite programs in countries that are not represented in this review. There were notably no documents from the continents of Asia and Africa. Unfortunately, good practices can go unreported in peer-reviewed publications; therefore, a review focusing on government reports and publications aimed at professionals could shed some light on promising respite models. From a practical point of view, this review serves as a starting point for the implementation of flexible home respite that is tailored to the caregivers’ and older adults’ needs. Our characterization of flexible at-home respite can be used to guide the improvement of existing respite services and to design new resources that reflect best practices in homecare, ultimately contributing to successful aging in place for older adults.

## Data Availability

The data supporting this study’s findings are available from the corresponding author, upon reasonable request.

## Abbreviations

PRISMAScR: Preferred Reporting Items for Systematic reviews and Meta-Analyses extension for Scoping Reviews
